# The novel role of ER protein TXNDC5 in the pathogenesis of organ fibrosis: mechanistic insights and therapeutic implications

**DOI:** 10.1186/s12929-022-00850-x

**Published:** 2022-09-02

**Authors:** Chen-Ting Hung, Yi-Wei Tsai, Yu-Shuo Wu, Chih-Fan Yeh, Kai-Chien Yang

**Affiliations:** 1 Department and Graduate Institute of Pharmacology, National Taiwan University College of Medicine, No. 1, Sec. 1, Ren-Ai Rd, 1150R, Taipei, 100 Taiwan; 2 Division of Cardiology, Department of Internal Medicine and Cardiovascular Center, National Taiwan University Hospital, Taipei, Taiwan; 3 Research Center for Developmental Biology & Regenerative Medicine, National Taiwan University, Taipei, Taiwan; 4 Center for Frontier Medicine, National Taiwan University Hospital, Taipei, Taiwan; 5 Department of Internal Medicine, National Taiwan University Hospital, Taipei, Taiwan; 6 Institute of Biomedical Sciences, Academia Sinica, Taipei, Taiwan

**Keywords:** Organ fibrosis, TXNDC5, Protein disulfide isomerase, Thioredoxin domain, Transforming growth factor-β (TGFβ)

## Abstract

Fibrosis-related disorders account for an enormous burden of disease-associated morbidity and mortality worldwide. Fibrosis is defined by excessive extracellular matrix deposition at fibrotic foci in the organ tissue following injury, resulting in abnormal architecture, impaired function and ultimately, organ failure. To date, there lacks effective pharmacological therapy to target fibrosis per se, highlighting the urgent need to identify novel drug targets against organ fibrosis. Recently, we have discovered the critical role of a fibroblasts-enriched endoplasmic reticulum protein disulfide isomerase (PDI), thioredoxin domain containing 5 (TXNDC5), in cardiac, pulmonary, renal and liver fibrosis, showing TXNDC5 is required for the activation of fibrogenic transforming growth factor-β signaling cascades depending on its catalytic activity as a PDI. Moreover, deletion of TXNDC5 in fibroblasts ameliorates organ fibrosis and preserves organ function by inhibiting myofibroblasts activation, proliferation and extracellular matrix production. In this review, we detailed the molecular and cellular mechanisms by which TXNDC5 promotes fibrogenesis in various tissue types and summarized potential therapeutic strategies targeting TXNDC5 to treat organ fibrosis.

## Introduction

Fibrosis, a non-physiological repair process in multiple organs, occurs in response to chemical, immunological and physical insults, evolutionarily designed as the body’s matrix synthetic machinery to repair and maintain tissue homeostasis. Upon injury, the release of multiple pro-fibrotic cytokines (such as transforming growth factor-β [TGFβ] and tumor necrosis factor-α [TNFα]), growth factors (such as connective tissue growth factor [CTGF], fibroblast growth factor 2 [FGF-2], insulin-like growth factor [IGF], platelet-derived growth factor [PDGF]) and reactive oxidants from damaged cells or infiltrating inflammatory cells triggers the activation and proliferation of fibroblasts [1–3]. Activated fibroblasts transition into α-smooth muscle actin (α-SMA)-expressing myofibroblasts, which are responsible for producing extracellular matrix (ECM) proteins that are required for acute tissue repair and wound healing [4]. This process is reversible once the tissue repair is completed, and activated fibroblasts are removed owing to apoptosis. If the injury to the tissue is perpetual, however, fibroblast activation becomes uncontrolled, turning themselves into apoptosis-resistant fibrosis-associated fibroblasts (FAFs). Incessant and excessive accumulation of FAFs and ECM proteins, therefore, leads to scar formation, architectural distortion, progressive loss of tissue function and ultimately organ failure.

TGFβ is the pivotal factor that drives organ fibrosis. Although TGFβ inhibits proliferation in most cell types and triggers apoptosis in epithelial cells, it stimulates mesenchymal cell proliferation and ECM production. TGFβ is secreted as part of a large latent complex, consisting of latent TGFβ-binding protein (LTBP), latency-associated peptide (LAP) and TGFβ itself [5]. After cleavage of LTBP and LAP by matrix metalloproteases or integrins, activated TGFβ dimer is released from the complex and interacts with TGFβ receptor 2 (TGFBR2) [6]. After binding with TGFβ, TGFBR2 recruits and phosphorylates TGFβ receptor 1 (TGFBR1), which further phosphorylates and activates SMAD2/3 to associate with SMAD4 [7]. The SMAD2/3/4 complex then enters nucleus and works with other co-transcription factors to regulate target genes to trigger the activation and trans-differentiation of resident or immigrated fibroblasts into myofibroblasts and ECM production. These activated myofibroblasts produce ECM proteins, such as collagens (Type I, III and IV), fibronectin, elastin and proteoglycan, to restore tissue integrity and maintain tissue homeostasis [8]. If the injury is not resolved properly, fibroblasts will continue to be activated to produce and accumulate excessive amounts of ECM proteins. This will consequently lead to the formation of stiff fibrotic matrix, where matrix stiffness provides mechanical stimulus to further promote fibrotic tissue remodeling and fibrosis progression [9]. In some cases, it could result in abnormal tissue architecture, function and ultimately organ failure. In addition to the canonical SMAD signaling pathway, TGFβ also stimulates non-canonical (SMAD-independent) pathways, such as mitogen activated protein (MAP) kinases (c-Jun N-terminal kinase [JNK], extracellular-signal regulated kinase [ERK] and p38), phosphatidylinositol-3-kinase (PI3K), Rho-like GTPases and janus kinases (JAKs) to induce organ fibrosis [10].

Fibrosis-related disorders cause enormous medical burden, and up to 45% of all deaths are attributed to severe fibrosis globally. However, current treatments for fibrosis have limited efficacy [11]. Of note, inhibiting the TGFβ signaling pathway directly to reduce organ fibrosis may cause undesired side effects. For example, blockage of TGFβ-mediated SMAD3 phosphorylation prevented bleomycin (BLM)-induced pulmonary fibrosis (PF); however, TGFβ or SMAD3 deletion caused abnormal lung organogenesis and systemic inflammation in mouse models [12, 13]. These evidence suggest that inhibition of TGFβ may cause systematic adverse effects and may not be an ideal therapeutic target for organ fibrosis. Delineating and leveraging novel mechanisms underlying organ fibrogenesis to develop potential therapeutic approaches, hence, are urgently needed [14]. Here, we discuss the current knowledge on the novel role of an endoplasmic reticulum (ER) protein thioredoxin (TRX) domain containing 5 (TXNDC5) in the pathogenesis of fibrosis, the underlying molecular/cellular mechanisms and the potential approaches to treat organ fibrosis by targeting TXNDC5.

## Thioredoxin domain containing 5 (TXNDC5)

**Fig. 1:**
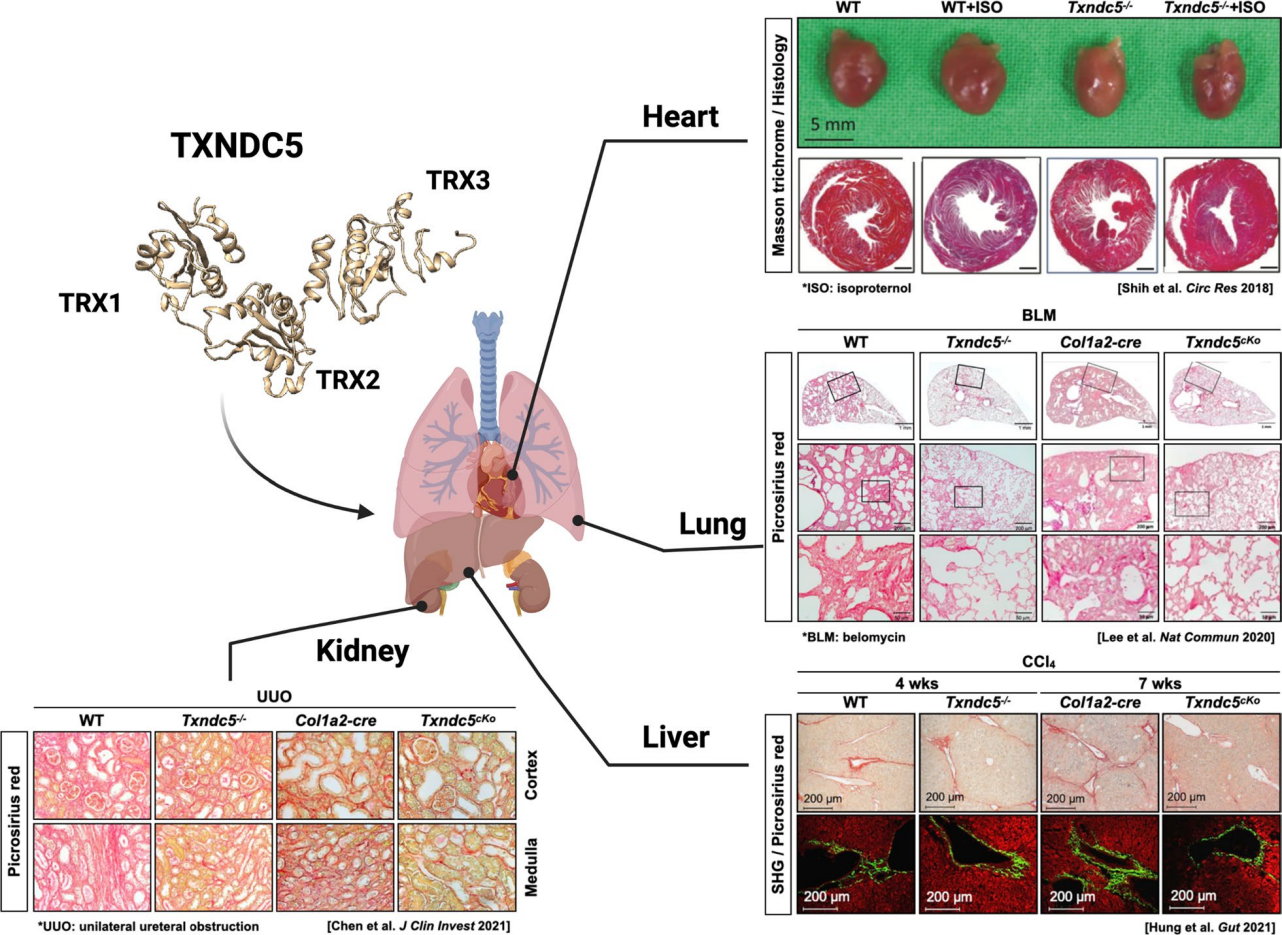
TXNDC5 contributes critically to the development of organ fibrosis through its PDI activity mediated by TRX domains. Global or targeted deletion of *Txndc5* prevents or halts fibrosis progression, as reflected by a reduction of fibrillar collagen deposition in internal organs including heart, lung, kidney and liver

TXNDC5, also known as endothelial protein disulfide isomerase (Endo-PDI), endoplasmic reticulum protein 46 (ERp46) or protein disulfide isomerase family A, member 15 (PDI15), is a member of PDI family. TXNDC5 catalyzes the formation of native disulfide bonds and rearranges the disulfide bonds via its TRX domains in the ER [15, 16]. Each TRX domain harbors a CGHC motif that serves as the catalytic domain for PDI activity (Fig. 1). Functionally, TXNDC5 facilitates proper protein folding, prevents unfolded protein response (UPR)-related apoptosis [15, 17], and mediates redox reaction via interacting with NADPH oxidase [18]. Moreover, TXNDC5 synergizes with heat shock cognate 70 protein (HSC70), another chaperone protein, to exacerbate the inflammatory phenotypes through NF-κB signaling, independent of its PDI activity [15, 19].

TXNDC5 was found enriched in endothelial cells (ECs) and fibroblasts [20], where TXNDC5 dysregulation was implicated in multiple diseases, including organ fibrosis, atherosclerosis, diabetes, liver disease, rheumatoid arthritis (RA), cancer, neurodegenerative disease and vitiligo [15, 21, 22]. In addition, TXNDC5 expression is induced under hypoxic conditions in disease states, including RA [23], non-small cell lung cancer [24], and colorectal cancer. A previous study demonstrated that TXNDC5 renders EC resistant to hypoxia-initiated apoptosis [20]. Taken together, current evidence suggests that aberrant TXNDC5 expression could contribute to a wide spectrum of diseases through its distinct functions in different cell types.

## The pathological role of TXNDC5 in organ fibrosis

TXNDC5 was reported to promote fibrosis in multiple organs, including the heart [25], lung [26], kidney [27], and liver [28], as the intermediary of TGFβ signaling (scheme shown in Fig. 1). TXNDC5 promotes tissue fibrosis through activation of canonical (SMAD3-dependent) or non-canonical (MAP kinases, such as JNK, ERK and pro-survival protein STAT3) TGFβ signaling. TGFβ induces TXNDC5 upregulation via increased ER stress level and activating transcription factor 6 (ATF6)-mediated transcriptional control. In addition, TRX domains of TXNDC5 contribute to the proper folding and stabilization of pro-fibrotic proteins. This TGFβ-ATF6-TXNDC5 signaling axis highlights the crucial role of TXNDC5 in fibrogenesis, although its downstream signaling mediators are slightly different in individual organs (shown in Table 1). In the following sections, we will describe the detailed mechanisms by which TXNDC5 mediates the development of organ fibrosis in heart, lung, kidney and liver.

**Table 1:** TGFβ-ATF6-TXNDC5 signaling axis triggers various downstream fibrogenic signaling pathways in different organs

Organ fibrosis	TXNDC5 downstream signaling pathway
Cardiac fibrosis [25]	1. Non-canonical TGFβ pathway: JNK signaling
	2. Facilitating folding of ECM protein, such as collagen and fibronectin
Pulmonary fibrosis [26]	1. Canonical TGFβ pathway: SMAD3 signaling
	2. Non-canonical TGFβ pathway: JNK and ERK signaling
	3. Post-translationally stabilizing TGFβR1 protein
Renal fibrosis [27]	1. Canonical TGFβ pathway: SMAD3 signaling
	2. Post-translationally stabilizing TGFβR1 protein
Liver fibrosis [28]	1. Non-canonical TGFβ pathway: JNK and STAT3 signaling

### The role of TXNDC5 in cardiac fibrosis (CF)

Heart failure (HF) is one of the major public health problems globally, with a rising prevalence and high mortality rate [29, 30]. Although medical advances reduce the mortality rates of cardiovascular diseases (CVD), including hypertensive heart disease, acute coronary syndrome, congenital and valvular heart diseases, it remains critical to develop novel treatment strategies for HF to reduce mortality rate [31, 32]. In addition to abnormalities in cardiomyocytes, CF contributes to cardiac remodeling and plays an important role in the progression of HF [33, 34]. Replacement of cardiac muscles by fibrotic tissues reduces systolic function following cardiac injury [33, 34]. Excessive ECM accumulation causes wall stiffness and results in diastolic as well as systolic dysfunction [33–35]. In addition to cardiac function, increased production of cardiac fibrotic tissues leads to enhanced cardiac automaticity and triggered activity, which can foster life-threatening arrhythmias [36, 37]. Targeting CF, therefore, may provide a novel therapeutic venue against contractile dysfunction, arrhythmias and death in HF.

During CF progression, renin–angiotensin–aldosterone system (RAAS), endothelin-1 (ET-1) and TGFβ1 expression levels are increased to trigger the activation and proliferation of fibroblasts [1–3]. Activated cardiac fibroblasts transdifferentiate into α-SMA-expressing myofibroblasts, secreting fibrillar collagen and multiple ECM proteins at the fibrotic foci [4]. Myofibroblasts also modulate ECM turnover through matrix metalloproteinases (MMPs) and tissue inhibitor of MMPs (TIMPs) to promote ECM accumulation during CF progression [38].

To date, therapeutic strategies against CF remain suboptimal. Using inhibitors targeting RAAS, including angiotensin converting enzyme inhibitors (ACEI), angiotensin receptor blockers (ARB) and mineralocorticoid receptor antagonist, has shown benefits in improving ventricular function and slow CF progression [39–41]. Nevertheless, these treatments cause a hypotensive effect which limited their capability to slow CF progression. Meanwhile, treatment using non-selective TGFβ inhibitors reduces fibrosis progression by attenuating fibroblast activation and ECM deposition in animal models [42–44]. However, these non-selective TGFβ inhibitors cause undesired side effects such as liver toxicity, limiting their clinical application [45]. Besides RAAS and TGFβ, therapeutic strategies targeting other fibrogenic molecules such as TGFβ activated kinase 1, p38, endothelin receptors, G protein-coupled receptor kinase 2 and miR21 have been investigated in preclinical studies [25, 46–50].

Recently, PDI family proteins have been implicated in multiple CVD [51]. Multiple studies have delineated the mechanisms by which PDIs contribute to the pathogenesis of CVD and shown the feasibility to treat these conditions by targeting PDIs [25, 52, 53]. Recently, Shih et al. reported that TXNDC5, or PDI15, regulates ECM accumulation and fibrosis progression [25]. TXNDC5 expression is upregulated in cardiac fibroblasts in human and mouse failing hearts with pathological cardiac hypertrophy. TXNDC5, a resident protein in the ER, facilitates ECM protein folding and activates cardiac fibroblasts via redox-sensitive JNK signaling pathway through its PDI activity. Deletion of TXNDC5 protects against isoproterenol-induced myocardial fibrosis, hypertrophy and contractile dysfunction in mice [25]. Because upregulation of TXNDC5 expression is restricted in activated cardiac fibroblasts, targeting TXNDC5, therefore, could inhibit CF with fewer undesired side effects than targeting TGFβ and RAAS.

### The role of TXNDC5 in pulmonary fibrosis (PF)

PF is a progressive clinical condition in which excessive buildup of fibrotic or scarring in the lung leads to the distorted pulmonary/alveolar structure, impaired lung function and gas exchange. Consequently, PF results in dyspnea, hypoxemia, exercise intolerance and ultimately death. Idiopathic PF (IPF), characterized by varying degrees of inflammation and scarring in the lung, is the most common type of interstitial lung diseases of unknown etiology. In Asia–Pacific countries, the adjusted incidence and prevalence of IPF ranged from 3.5 to 13 and 5.7 to 45.1 cases per 100,000 population, respectively [54]. Exploiting large population-based database of the Taiwan National Health Insurance, the accumulative prevalence rates increased steadily from 3.1 to 6.4 cases per 100,000 people per year during 2006–2011 based on a narrow case definition. More importantly, the mean survival time after IPF diagnosis is 6.9 year [55]. PF has become a huge economic and clinical burden globally. The therapeutic approaches for PF remain suboptimal to improve the quality of life and even increase survival due to insufficient understanding of pathogenetic mechanisms of PF [56]. Therefore, there is an urgent need to develop the novel therapies to improve the outcomes of PF patients.

The complex pathophysiological mechanisms including genetic predisposition and injury types may account for the initiation and progression of PF. In general, fibrosis is resulted from repetitive injury in alveolar epithelial cells by exogenous (e.g., infection, toxin and radiation) or endogenous (e.g., inflammation, oxidative stress and aberrant immune responses) stimuli. Increased profibrotic and inflammatory cytokines, including TGFβ, TNFα, CTGF, PDGF, etc., induce a series of signaling cascades to initiate the activation and proliferation of lung fibroblasts [57]. Activated fibroblasts are transdifferentiated into collagen-secreting myofibroblasts, which promote the production of fibrogenic ECM proteins and cause an imbalance between MMPs and its inhibitors, TIMPs, leading to excessive ECM accumulation at fibrotic foci and resulting in abnormal lung structure [58]. In addition, ER stress increases in response to lung injury, triggering myofibroblast transdifferentiation and epithelial–mesenchymal transition, both of which can contribute to PF [59, 60].

Immunosuppressive and immunomodulatory therapies, such as a combination of prednisone, azathioprine and *N*-acetylcysteine, exhibit limited therapeutic effects in terms of mortality and hospitalization [61, 62]. In addition, targeting pro-fibrotic cytokines and chemoattractants, such as etanercept, a recombinant soluble human TNF receptor, and carlumab, a CC-chemokine ligand 2 monoclonal antibodies, also fails to improve IPF outcomes [63, 64]. Currently, two drugs, pirfenidone (5-methyl-1-phenylpyridin-2[1*H*]-one) and nintedanib, are utilized to treat IPF. Pirfenidone, a synthetic compound of phenyl pyridine, inhibits the TGFβ signaling and other inflammatory cytokines (e.g., TNFα and IL-1β) [65, 66]. Clinical trials revealed that pirfenidone delays disease progression and prevents deterioration of pulmonary function in IPF patients [67–69]. Exploring pooled analyses and meta-analyses of clinical trials, pirfenidone was shown to provide outcome benefits [70]. Nintedanib, a tyrosine kinase inhibitor, targets vascular endothelial growth factor receptor, FGF receptor and PDGF receptor. By suppressing the kinase activities, nintedanib also slows down disease progression of IPF, although mild to moderate diarrhea and nausea are observed in most cases [71]. Collectively, both agents show the potential to attenuate PF through inhibition of inflammation. However, these two agents are not able to stop the progression of IPF and there is an urgent need to develop newer therapies to improve the outcome of IPF patients.

Recent study by Lee et al. demonstrated that TXNDC5 is involved in the progression of IPF through modulating TGFβ signaling [26]. In this study, the transcript and protein expression of TXNDC5 are upregulated in the lung tissues of human IPF patients and mice with BLM-induced lung fibrosis. Mechanistically, TGFβ1 stimulation induces TXNDC5 upregulation in lung fibroblasts via increased ER stress levels and ATF6-mediated transcriptional regulation. Consequently, both TGFβ canonical- (SMAD3) and non-canonical (JNK and ERK) signaling are activated, resulting in the activation, transdifferentiation, proliferation and ECM production in lung fibroblasts. TXNDC5 enhances the protein stability of TGFBR1, but not TGFBR2, leading to amplification of TGFβ signaling. Importantly, forced expression of mutant TXNDC5 (Cys-to-Ala mutations at the both ends in the CGHC motif of the three TRX domains) in lung fibroblasts inhibits PDI activity, lowers the protein expression of TGFBR1 and consequently inactivates its downstream signaling pathways. Collectively, TXNDC5 stabilizes TGFBR1 and augments TGFβ signaling, generating a positive feedback loop of TGFβ1-ATF6-TXNDC5-TGFBR1 signaling axis to cause severe scarring in the lung [26]. In addition, global deletion of *Txndc5* protects against BLM-induced PF and impairment of pulmonary function without altering inflammatory response to BLM. Utilizing inducible fibroblast-specific deletion of *Txndc5* further confirms the pathogenic requirement of fibroblastic TXNDC5 in the development and progression of lung fibrosis, thereby preventing and even reversing pulmonary dysfunction in BLM-treated animals. Taken together, these results show that TXNDC5 modulates TGFβ signaling activity during the development of lung fibrosis and highlight the therapeutic potential to treat patients with PF by targeting TXNDC5 in lung fibroblasts.

### The role of TXNDC5 in renal fibrosis (RF)

Chronic kidney disease (CKD) is caused by chronic and progressive injuries to kidney tissue. The rising prevalence of metabolic diseases, inflammation, hypertension and obesity, as well as aging increases the risk of kidney injury [72]. CKD affects more than 10% of the population worldwide, with an estimated prevalence of 11.7 to 15.1%. Advanced CKD can turn into end-stage renal disease (ESRD). Patients with ESRD requiring dialysis or kidney transplantation are estimated to be between 4.902 and 7.083 million, representing a substantial clinical and socioeconomic burden [73]. Moreover, Taiwan has the highest incidence of CKD (estimated prevalence of 15.46%) in Asia [74]. In order to repairing the damaged tissues by wound healing, fibrogenic proteins are produced in response to kidney injury, resulting in excessive accumulation of fibrogenic proteins at fibrotic area and consequently RF. Glomerulosclerosis and tubulointerstitial fibrosis are the most common types of fibrosis observed in CKD.

Dysregulation of RAAS plays a major pathological role in CKD-induced fibrosis [75]. Angiotensin II triggers the release of pro-fibrotic factors, leading to renal inflammation and fibrosis [76, 77]. The standard therapy to delay the progression of CKD is blockade of RAAS using ACEIs, ARBs, or direct renin blockers. Among antihypertensive agents, both ACEIs and ARBs are considered as the feasible approaches against RF, including lowering blood pressure, reducing proteinuria, ameliorating RF and delaying the progression of CKD [77–79]. RAAS blockade, however, is not sufficient to halt the progression of CKD in many cases. In addition, the endothelins, especially ET-1, promote RF through increasing renal vasoconstriction and glomerular pressure [80]. In a preclinical study, ET-1 receptor A (ET_A_) antagonist, ABT-627, has shown to prevent glomerulosclerosis, attenuate vascular fibrosis and collagen deposition in hypertensive rats [81, 82]. However, two clinical trials raised the safety concerns using ET_A_ antagonists, including significant fluid overload and congestive HF, in patients with renal diseases [83, 84].

TGFβ, as shown in the previous sections, is known to promote fibrosis in most, if not all, organs, and thereby numerous therapeutic are designed to target TGFβ or its downstream signaling. The humanized anti-TGFβ antibody, LY2382770, is designed to neutralize TGFβ, failed to show therapeutic efficacy against CKD [85]. Pentoxifylline, a nonspecific phosphodiesterase inhibitor, attenuates tubulointerstitial fibrosis in CKD animal model (unilateral ureteral obstruction [UUO]) by interfering with the transcription of SMAD3/4 to suppresses CTGF [86]. Pentoxifylline was shown to improve renal function in high-risk patients, however, this study lacks its power due to small sample size and incomplete follow-up [87]. Directly targeting fibrogenic proteins such as CTGF is an alternative approach. Indeed, reducing CTGF expression levels by antisense oligonucleotide (ASO) significantly attenuates the progression of CKD and fibrosis in mice subjected to UUO surgery [88]. Even though intravenous administration of CTGF antibody (FG-3019) in patients with diabetic kidney disease significantly decreases albuminuria without obvious adverse effects, the safety concerns, such as the interference with CTGF-dependent skeletogenesis, still exist as CTGF is involved in more complex biological processes, such as angiogenesis, chondrogenesis and osteogenesis. [89, 90]. Finally, pirfenidone, approved by US Food and Drug Administration (FDA) to treat IPF, also exhibits anti-fibrotic effects in the RF animal models by suppressing the mesangial matrix expansion [91]. However, the therapeutic effects of pirfenidone in RF patients are inconsistent, requiring more clinical trials to prove its efficacy [92, 93].

A recent study by Chen et al. [27] demonstrated that TXNDC5 modulates TGFβ/ATF6/TGFβR1 signaling axis in RF, similar to that observed in PF [26]. The transcript and protein expressions of TXNDC5 are upregulated in the kidneys of CKD patients and mice, especially in collagen-secreting kidney fibroblasts. The ATF6-dependent ER stress pathway transcriptionally regulates TXNDC5 in fibroblasts following TGFβ stimulation. Depletion of TXNDC5 attenuates in human kidney fibroblasts activation, proliferation and ECM production induced by TGFβ1. Forced TXNDC5 expression not only triggers human kidney fibroblasts activation, proliferation and ECM production but also augments TGFβR1 protein stabilization to activate TGFβ canonical signaling pathway, resulting in a “positive feedback loop”. However, such phenotypes are abolished in overexpressing catalytically dead TXNDC5 with mutant TRX domains. In addition, targeting TXNDC5 in kidney fibroblast attenuates the extent of scarring in multiple RF mouse models, including UUO, unilateral ischemia–reperfusion injury and folic acid-induced RF. Deletion of *Txndc5* in other kidney cell types, including renal tubular epithelial cells, podocytes and ECs, however, has no obvious impact on fibrosis progression. More importantly, inducing deletion of *Txndc5* in kidney fibroblasts in animals with existing UUO-induced RF completely halted the progression of fibrosis and preserves kidney function. Taken together, these results illustrate that targeting TXNDC5 in renal fibroblasts attenuates the progression of RF by breaking down the positive feedback loop of TGFβ/ATF6/TGFβR1 signaling axis.

### The role of TXNDC5 in liver fibrosis (LF)

LF is caused by chronic liver injuries [94], including viral infection, alcohol use, non-alcoholic steatohepatitis (NASH) and obstructive biliary diseases including primary biliary cholangitis, primary sclerosing cholangitis and biliary atresia [95, 96]. Chronic hepatocellular injury leads to damage of epithelial/endothelial barrier, release of inflammatory cytokines and recruitment of inflammatory cells, followed by the secretion of pro-fibrotic cytokines. Hepatic myofibroblasts are then activated to produce excessive ECM proteins for the formation of fibrous septae and regeneration nodules [96, 97]. Myofibroblasts originate from hepatic resident cells, including hepatic stellate cells (HSCs) and portal fibroblasts, or bone marrow-derived cells, including fibrocytes and mesenchymal stem cells [98–101]. Although multiple cell types contribute to ECM production, HSC is a major source of ECM and contributes to the pathogenesis of almost all types of LF [102].

In normal liver, quiescent HSCs reside in the space of Disse between hepatocytes and liver sinusoidal endothelial cells, function as pericytes and are the major storage sites of vitamin A [103–105]. These HSCs contribute to one-third of non-parenchymal cells in the liver and exhibit a non-proliferative, quiescent phenotype in normal liver [103, 106, 107]. In response to liver injury, damaged hepatocytes and inflammatory cells secret fibrogenic cytokines, including TGFβ, TNFα, CTGF, etc., and reactive oxidants, which activate and transform quiescent HSCs into highly proliferative myofibroblasts [103].

LF can be reversible if the underlying causes of liver injury are removed in its early stages [108]. For example, antiviral therapy is associated with reduced Child–Pugh scores in viral hepatitis caused by hepatitis B virus or hepatitis C virus [109, 110]. Abstinence from alcohol also shows great efficacy of restoring liver function in alcohol-related liver disease [111]. For NASH induced by overweight and obesity, reducing body weight through dietary and lifestyle modification is effective to treat NASH [112–114]. If liver damage reaches irreversible stages and end-stage cirrhosis ensues, liver transplantation remains the only curative treatment. To date, therapies directly targeting LF remain unavailable. Importantly, therapies targeting underlying sources of liver injury, such as antiviral therapy against hepatitis B virus infection, are expensive and may require lifelong medication. Moreover, there is no approved medication for NASH so far [108, 113]. Therefore, therapies to reduce HSC activation and ECM accumulation directly emerge as a new approach to treat LF and liver cirrhosis.

The role of ER protein TXNDC5 has been recently studied in the progression of LF [28]. Hung et al. demonstrated TXNDC5 is considerably expressed in activated HSCs and at fibrotic foci of the livers from human patients and mice with liver fibrosis/cirrhosis. TXNDC5 induces HSC activation through reactive oxygen species (ROS)-dependent JNK signaling; TXNDC5 also renders HSCs resistant to apoptosis via STAT3 signaling, leading to accumulation of activated HSCs and excessive fibrotic scar in the liver. Inhibiting the catalytic function of TXNDC5 abolishes JNK and STAT3 activation and the downstream fibrotic responses. Intriguingly, targeted ablation of *Txndc5* in HSCs, but not hepatocytes, significantly protects against the development and progression of LF in mice with hepatotoxic (CCl_4_ treatment) or cholestatic (bile duct ligation) injury, as evidenced by a lower fibrillar collagen deposition and preservation of liver function. Taken together, targeting TXNDC5 may build a new avenue for liver fibrosis/cirrhosis treatments via blocking HSC activation, proliferation, ECM production, as well as depriving anti-apoptotic capacity of HSCs.

Upon acute or chronic liver injury, pro-fibrotic cytokine TGFβ triggers activation of ER stress pathway. ATF6-p50, an active form of ATF6, translocates into the nucleus from the cytoplasm to physically interact with the promoter of TXNDC5, leading to*TXNDC5* upregulation. Increased TXNDC5 expression leads to transdifferentiation of HSCs into myofibroblasts, resulting in considerable myofibroblast proliferation and ECM production. These responses depend on the redox-activity of TXNDC5 to trigger TGFβ canonical and non-canonical signaling and stabilize ECM and TGFβR1 proteins, leading to a positive feedback loop of TGFβ/ATF6/TGFβR1 signaling.

**Fig. 2:**
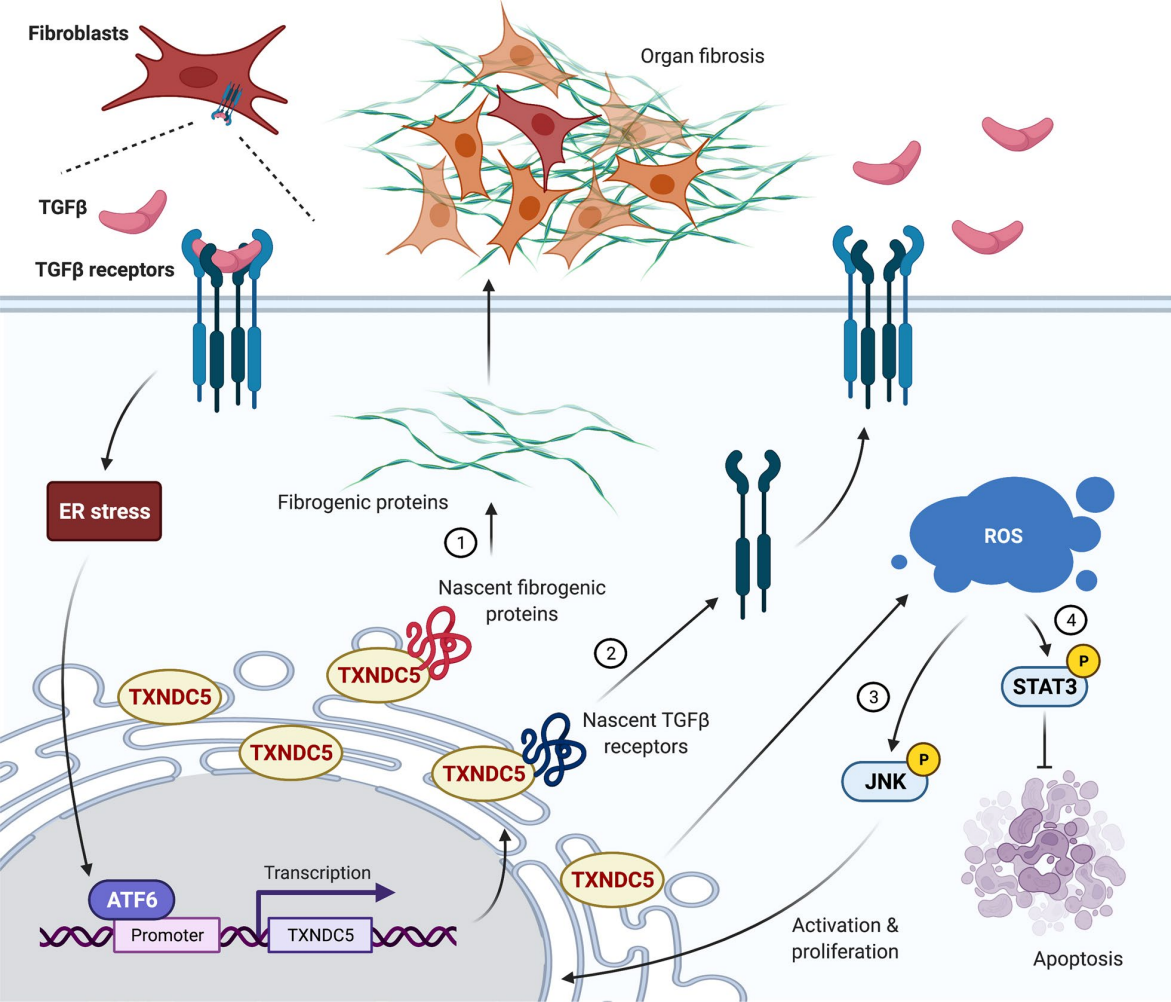
Summary of the molecular mechanisms by which TXNDC5 promotes organ fibrogenesis. Schematic illustration of the mechanisms by which TXNDC5 contributes to organ fibrosis. TGFβ-stimulated ER stress activates the ATF6 branch, which transcriptionally activates TXNDC5 by physically interacting with TXNDC5 promoter. Increased TXNDC5 levels promote fibrogenic responses through 4 context-dependent mechanisms including (1) facilitating proper folding of fibrogenic ECM proteins, (2) stabilizing TGFβ receptor 1, and activating TGFβ non-canonical (3) JNK and (4) STAT3 signaling

Taken together, these studies suggest that TXNDC5 is a critical yet previously unrecognized mediator of organ fibrosis. TXNDC5 mediates organ fibrosis through 4 distinct, context-dependent mechanisms: (1) facilitating ECM protein folding, including collagen and fibronectin; (2) stabilizing TGFBR1 protein; (3) triggering TGFβ non-canonical JNK signaling to induce fibroblast activation and proliferation; (4) activating phosphorylated STAT3 to render fibroblasts resistant to apoptosis. Figure 2 summarizes the detailed mechanisms by which TXNDC5 promotes organ fibrosis.

## The role of TXNDC5 in other fibrosis-related diseases

Because fibroblasts are a major cellular constituent in almost all tissues, fibrosis can be involved in the dysfunction of multiple organs. RA, for example, is a chronic inflammatory disease characterized by hyperplasia of synovial fibroblasts, causing progressive joint destruction, chronic synovitis and consequently functional disability [115]. Clinical studies indicated that treatment begins early with medications that remises symptoms. In general, medications for RA includes anti-inflammatory drugs, such as nonsteroidal anti-inflammatory drugs (NSAIDs) and steroids, and relieves symptoms drugs, such as methotrexate, leflunomide, hydroxychloroquine, etc. Previously, Wang et al. reported that TXNDC5 is detected in synovial tissues and blood from RA patients [116]. Increased TXNDC5 results in abnormal proliferation and migration of synovial fibroblasts, which are detected in the joints of the toe, knee and the ankle, in TXNDC5 transgenic mice following collagen-induced arthritis. Moreover, hypoxia induces synovial fibroblasts proliferation, migration and TXNDC5 expression, whereas reducing TXNDC5 inhibits these responses. Additionally, TXNDC5 has been reported to synergize with HSC70 to exacerbate the inflammatory phenotype of synovial fibroblasts via activating NF-κB signaling by destabilizing IκBβ protein [19]. Collectively, targeting TXNDC5 might be a potential therapy to reduce joint destruction and synovitis in RA patients.

## Therapy approaches and clinical applications to target TXNDC5

Currently, pirfenidone, is the only anti-fibrotic agent approved by FDA for treating IPF via, at least partially, suppressing TGFβ signaling [117]. However, TGFβ is an important growth factor that controls many cellular responses, including proliferation, differentiation, etc., and implicates in the development and homeostasis of most human tissues. Therefore, inhibition of TGFβ could result in unexpected and harmful side effects. Leveraging the abovementioned pathological mechanism, targeting ER stress and PDI, particularly TXNDC5, offers a new way to design anti-fibrotic drugs. We will discuss some potential strategies to target TXNDC5 as the treatment of organ fibrosis.

### Inhibition of ER stress response to repress TXNDC5 expression

4-Phenylbutyric acid (4-PBA) is an US FDA-approved drug and currently used for the treatment of urea cycle disorders. 4-PBA is metabolized through β-oxidation to phenylacetate and then conjugated to glutamine to form phenylacetylglutamine, which is excreted by the kidneys [118, 119]. Mechanistically, it inhibits the aggregation of misfolded proteins and mitigates ER stress, suggesting its potential to treat fibrosis [120]. Indeed, recent studies demonstrated that treatment with 4-PBA significantly downregulates fibrosis-related genes (TGFβ1, phosphor-SMAD2 and pro-collagen isoform) induced by pressure overload, prevents the activation of UPR and decreases collagen deposition, halting the development of CF and adverse remodeling [121, 122]. Additionally, blocking ER stress by 4-PBA treatment successfully attenuates UUO-induced kidney fibrosis in rats, as reflected by the lower expression of pro-fibrotic proteins (collagen type 1α, fibronectin and α-SMA) [123]. In addition to the evidence mentioned above, pre-treatment with 4-PBA reduces TGFβ-induced TXNDC5 expression in the human fibroblasts from multiple organs, including the heart, lung, kidney, and liver, suggesting its potential use to repress TXNDC5 expression caused by TGFβ-induced ER stress, thereby attenuating organ fibrosis [25–28].

Tauroursodeoxycholic acid (TUDCA), a hydrophilic bile acid, is one of the chemical chaperons and is approved by FDA for use in biliary cirrhosis and cholestatic liver diseases based on its choleretic effects [124]. Recent studies have demonstrated that TUDCA might be therapeutic in several diseases, including neurodegenerative diseases, osteoarthritis, vascular diseases and diabetes [125–128]. Functionally, TUDCA ameliorates ER stress and prevents UPR dysfunction in part by improving protein folding capacity and by supporting the transfer of mutant proteins [129]. A previous study revealed that TUDCA serves as an inhibitor of apoptosis by negatively regulating the mitochondrial pathway of cell death, reducing ROS production and inhibiting apoptosis associated with ER stress [130]. In addition, treatment with TUDCA, in human cardiac fibroblasts, leads to a reduction of TGFβ-induced TXNDC5 expression via blocking ER stress, thereby attenuating cardiac fibrogenesis in mice [25].

Collectively, blocking ER stress could be an effective strategy to inhibit TXNDC5 expression in fibroblasts, thereby inhibiting fibrogenesis. However, upregulation of ER stress and UPR activation enhances not only the capacity of protein folding and maturation but also protein degradation and transport pathway, thereby alleviating the burden of misfolded protein [131]. Therefore, overt suppression of ER stress may result in an imbalance of homeostasis, and consequently causing undesired side effects.

### Functional inhibition of TRX domains of TXNDC5

The PDI-activity mediated by the TRX domains is essential for TXNDC5 to trigger ROS production and stabilizing fibrogenic proteins. Therefore, therapeutic agents that target TRX domains can inhibit PDI activity of TXNDC5, leading to attenuated fibrogenic responses. An irreversible PDI activity inhibitor, 16F16, covalently binds to the active site of cysteines and has been shown to prevent oxidation of the targeted protein, suppress misfolded proteins-induced apoptosis and protect against neurodegenerative disorders caused by accumulation of misfolded proteins [132, 133]. In addition, 16F16 inhibits functional activity of TXNDC5 via blocking TRX domains, leading to inhibition of redox-sensitive pro-fibrotic signaling pathways of TXNDC5 [25, 28]. However, 16F16 is a non-selective PDI inhibitor because it also targets TRX domains in other PDI family proteins. Some PDIs are known to be essential for normal physiological functions, such as regulation of calcium release in muscle [134], and the development of the mucus-secreting cement gland [135]. Developing a TXNDC5-specific PDI inhibitor could be a powerful way to treat fibrosis without the risk of disturbing the homeostasis in non-fibroblast cells and other organ functions.

### Targeted deletion of TXNDC5 by genetic targeting therapies

Although the aforementioned in vitro and in vivo evidence have shown targeting TXNDC5 in mouse fibroblasts successfully halts the progression of fibrosis in multiple organs, it remains challenging to design applicable therapeutic strategies to specifically target TXNDC5 in human fibroblasts. Numerous genetic targeting therapies, including small interfering RNA (siRNA), inhibitory ASO, etc., have currently charted into clinical trials and been approved to treat diseases [136]. For example, the first FDA-approved double-stranded siRNA therapeutics, patisiran (ONPATTRO™), encapsulated in lipid nanoparticles for delivery to hepatocyte, is used to treat the polyneuropathy of hereditary transthyretin (TTR)-mediated amyloidosis (hATTR) in adults [137]. In addition, Inotersen, an ASO inhibitor of the hepatic production of transthyretin protein, is also approved by FDA to treat hATTR [138]. Designing TXNDCX5-targeting siRNA or specific ASO to target its TRX domain could be useful to treat fibrotic diseases by repressing TXNDC5 expression and interfering with its PDI activity.

Clustered regularly interspaced short palindromic repeats (CRISPR) and CRISPR-associated 9 protein (CRISPR/Cas9) system is a revolutionary gene-editing technology and has been extensively exploited in biomedical research and clinical investigation [139, 140]. CRSIPR/Cas9 complex comprised by a single guild RNA and Cas9 protein, leading to double-stranded breaks at anchored site of the target gene. Yeh et al. has recently utilized nanoparticles carrying an endothelium-specific *Txndc5*-targeting CRISPR/Cas9 vectors to specifically delete endothelial TXNDC5, which effectively ameliorates disturbed blood flow-induced carotid atherosclerosis [22]. CRISPR/Cas9 therapy had also been approved by FDA to treat certain genetic diseases in human patients, such as correcting the mutation of a β-globin gene in the sickle cell disease [141].

Taken together, therapeutic strategies using RNAi, ASO and CRISPR/Cas9 system encapsulated by nanoparticles to delete fibroblast-specific TXNDC5 could be a potential therapeutic approach against organ fibrosis in the future.

## Conclusions

Emerging evidence has demonstrated the essential role of TXNDC5 in organ fibrogenesis by inducing fibroblast activation, proliferation and ECM production through its PDI activity. TXNDC5 forms a complex regulatory network to amplify TGFβ-induced fibrogenic response via folding/stabilizing ECM and TGFβR1 proteins. More importantly, therapeutic strategies to target TXNDC5 have unique advantages due to its fibroblast-restricted expression pattern and the fact that it’s non-essential for physiological function. Therefore, novel small molecules, gene-editing approaches, siRNA or ASO that target TXNDC5 could be a powerful approach to treat or prevent organ fibrosis and preserve organ function.

## Data Availability

Not applicable.

## Abbreviations

4-PBA: 4-Phenylbutyric acid
ATF6: Activating transcription factor 6
ACEI: Angiotensin converting enzyme inhibitors
ARB: Angiotensin receptor blockers
ASO: Antisense oligonucleotide
BLM: Bleomycin
CF: Cardiac fibrosis
CKD: Chronic kidney disease
JNK: c-Jun N-terminal kinase
CRISPR: Clustered regularly interspaced short palindromic repeats
CTGF: Connective tissue growth factor
CRISPR/Cas9: CRISPR-associated 9 protein
ER: Endoplasmic reticulum
ERp46: Endoplasmic reticulum protein 46
ECs: Endothelial cell
Endo-PDI: Endothelial protein disulfide isomerase
ET-1: Endothelin-1
ETA: Endothelin-1 receptor a
ESRD: End-stage renal disease
ECM: Extracellular matrix
ERK: Extracellular-signal regulated kinase
FGF-2: Fibroblast growth factor 2
FAFs: Fibrosis-associated fibroblasts
FDA: Food and Drug Administration
HSC70: Heat shock cognate 70 protein
HSCs: Hepatic stellate cells
IPF: Idiopathic pulmonary fibrosis
IGF: Insulin-like growth factor
JAKs: Janus kinases
LAP: Latency-associated peptide
LTBP: Latent TGFβ-binding protein
LF: Liver fibrosis
MMPs: Matrix metalloproteinases
MRA: Mineralocorticoid receptor antagonist
MAP: Mitogen activated protein
NSAIDs: Nonsteroidal anti-inflammatory drugs
PI3K: Phosphatidylinositol-3-kinase
PDGF: Platelet-derived growth factor
PDI: Protein disulfide isomerase
PDI15: Protein disulfide isomerase family a, member 15
PF: Pulmonary fibrosis
ROS: Reactive oxygen species
RF: Renal fibrosis
RAAS: Renin–angiotensin–aldosterone system
siRNA: Small interfering RNA
TUDCA: Tauroursodeoxycholic acid
TGFβ: Transforming growth factor-β
TGFBR1: TGFβ receptor 1
TGFBR2: TGFβ receptor 2
TRX: Thioredoxin
TXNDC5: Thioredoxin domain containing 5
TIMPs: Tissue inhibitor of MMPs
TTR: Treat the polyneuropathy of hereditary transthyretin
hATTR: Treat the polyneuropathy of hereditary transthyretin-mediated amyloidosis
TNFα: Tumor necrosis factor-α
UPR: Unfolded protein response
UUO: Unilateral ureteral obstruction
α-SMA: α-Smooth muscle actin
